# Cancer Doctors

**Published:** 1870-07

**Authors:** 


### "Cancer Doctors."

### HOW THEY SWINDLE THE PEOPLE—DANGER OF THEIR PLASTERS.

We were recently called upon to amputate a breast that had become cancerous by reason of the irritation produced through the application of a cancer plaster, applied to a harmless spot (nævus) upon the surface of a healthy lady's breast. So frequently have we been called upon to interfere in cases of this sort, that we have been persuaded in our mind to warn our readers against the practice of these so-called "cancer doctors."

The lady referred to was called upon by one of those ignorant itinerants, who is in the habit of pronouning every mole, nævus, scar or mark upon the surface of the body a cancer. She heard his recital of the danger of cancer—how hundreds die from their horrible effects—how imperceptibly they make their appearance—and how wonderful his cancer-plaster was in effecting a cure. Her mind was so influenced by his terrible story, that she persuaded herself that a harmless, red mark upon her breast, might be one of those fearful cancers. So, she exhibited it to the knowing cancer doctor, who at once pronounced it a "spider cancer,"—showed her the legs of the spider—explained how these legs would become deep seated roots that would dip down into her flesh and destroy her life; congratulated her upon his opportune arrival, and at once gained her confidence and consent to apply his wonderful plaster. It was done. The plaster, composed principally of arsenic, destroyed the skin, eat deeply into the flesh, and established a painful and ugly sore, which all the skill of the Cancer Doctor failed to heal. The disease continued until a vascular, bleeding growth, as large as a tea-cup, made its appearance upon the surface of her breast. She became frightened, applied to a physician, who told her she had indeed a cancer, (*fungus hæmatodes*,) and advised an immediate removal of the entire breast. She applied to us for that purpose, and recited the above particulars to us.

Most persons who chance to have some slight discoloration of the skin upon any portion of the body, are apt to imagine that it is cancerous in its nature. People seem to be monomaniacs upon the subject of cancers, if we may judge by the numbers who apply to us, demanding examinations of all manner of little trifles, in the way of marks, moles, warts, and those little red spots that appear like a net-work of small vessels under the skin. All these are magnified into cancers, and our advice is sought as to the best means of disposing of them. We are glad they come to us, for we can then set their minds at rest, and warn them against the visitations of the Cancer Doctor with his everlasting plaster.

Sometimes these fellows succeed in removing little *encysted tumors*, located under the skin, with their plaster, which can be done with safety, often, when not located near any important gland like the breast. When they do succeed, they at once secure a certificate from their over-credulous victims, of a "wonderful cure of cancer," which is at once proclaimed through the medium of the village paper, to those similarly afflicted. But, let me tell you, that while you dispose of a harmless little tumor, which, if an annoyance to you, could be removed by a surgeon without pain or danger, you are absolutely running a risk of your life at the hands of an ignoramus who is not competent to tell when you are in danger, or how to relieve you when such danger is imminent. Aviod them as you would the small pox—hide from them as you would from a book-peddler—shun them as you would the devil, for they will not only swindle you out of your money, but your life as well.

We have seen their work, and know whereof we affirm. If you have a mark upon your body that frightens you, go to your family physician with it; he is certainly more competent to judge whether it is a cancer or not than one of these self-constituted cancer doctors; if you doubt his ability in such cases, there are skillful surgeons to be found in every city, apply to them; but arm yourself with a broom, if a woman—with a boot jack if a man—and drive every rascally cancer-doctor from your door, and be spared such suffering as we have just narrated.

GAY AND FESTIVE.—We see an announcement in a California paper that Gen. Sheridan says, that the Santee Indians sing Watt's hymns while engaged in their scalp dance. The idea is a poetical one. Just imagine a gay and festive brave, full of fire-water, driving a tomahawk through the skull of a pale-faced prisoner, and then, after building a fire upon his chest, waltzing around singing, "I want to be an angel!" These simple children of the forest have immortal longings as well as a great weakness for hymns.

